# Draft Genome Sequence of Vibrio penaeicida Strain TUMSAT-NU1, Isolated from Diseased Shrimp in Japan

**DOI:** 10.1128/genomeA.00153-18

**Published:** 2018-03-15

**Authors:** Satoshi Kawato, Reiko Nozaki, Hidehiro Kondo, Ikuo Hirono

**Affiliations:** a Laboratory of Genome Science, Tokyo University of Marine Science and Technology, Tokyo, Japan

## Abstract

Vibrio penaeicida is a bacterial pathogen of cultured shrimp. The draft genome sequence of V. penaeicida strain TUMSAT-NU1 consists of 100 scaffolds with a total of 6.41 Mbp. We identified possible virulence factors, and we found that V. penaeicida and Vibrio nigripulchritudo are closely related.

## GENOME ANNOUNCEMENT

The Japanese shrimp aquaculture industry has experienced massive economic losses caused by various infectious diseases (1). Vibriosis is one of the most serious bacterial infections in shrimp aquaculture, and researchers have made substantial efforts to characterize shrimp-pathogenic Vibrio spp. (1).

Vibrio penaeicida has long been known as a shrimp pathogen in Japan and New Caledonia (2–6). The first reported outbreak of V. penaeicida dates back to 1982 in Japanese shrimp ponds (2). V. penaeicida was also responsible for a vibriosis outbreak in New Caledonia in 1993 (6). Biochemical and molecular analyses established that V. penaeicida represents a distinct species of the genus Vibrio (3, 4); however, knowledge on the V. penaeicida genome has been lacking.

Here, we present the draft genome sequence of V. penaeicida strain TUMSAT-NU1 (5), which was originally isolated from a diseased shrimp in the 1980s in Japan. We extracted the bacterial genomic DNA using the standard cetyltrimethylammonium bromide (CTAB) method and constructed a paired-end DNA library with a Nextera XT library preparation kit (Illumina, USA). We sequenced the library with the MiSeq platform and MiSeq reagent kit version 2 (300 cycles). We assembled the read data using CLC Genomics Workbench 6.5.2 (Filgen, USA) and annotated the resulting contigs on the Rapid Annotations using Subsystems Technology (RAST) server (7–9). To search for bacterial genomes that are closely related to V. penaeicida TUMSAT-NU1, we used NCBI BLAST with several V. penaeicida TUMSAT-NU1 scaffolds as queries against the NCBI nonredundant nucleotide database. Finally, we used Genome-to-Genome Distance Calculator (GGDC) 2.1 (http://ggdc.dsmz.de/ggdc.php) (10) for *in silico* species delineation by comparing the V. penaeicida TUMSAT-NU1 genome and the V. nigripulchritudo reference genome (GenBank accession numbers NC_022528 and NC_022543).

*De novo* assembly of paired-end read data resulted in 100 scaffolds with a total of 6.41 Mbp. The RAST pipeline identified 5,869 features, including dozens of putative protease genes, some of which might be the virulence factor(s) that affect penaeid shrimp (11). Moreover, genes related to type I, II/IV, and VI secretion systems were present, suggesting that they serve as toxin secretion machinery in V. penaeicida.

A preliminary search with NCBI BLAST revealed that V. penaeicida TUMSAT-NU1 is closely related (80% to 90% nucleotide identity) to V. nigripulchritudo, another shrimp-infecting Vibrio (12). Analyses on the GGDC website yielded an estimated DNA-DNA hybridization value of 21.20% and a G+C content difference of 1.89%, indicating that the two strains represent distinct species. These results demonstrate that V. penaeicida TUMSAT-NU1 is closely related to, but different from, V. nigripulchritudo and suggest that these two organisms comprise a distinct group of shrimp-associated bacteria in the genus Vibrio.

Here, we reported on the draft genome sequence of V. penaeicida TUMSAT-NU1. Our results will contribute to a deeper understanding of the molecular basis of bacterial infections in cultured shrimp.

### Accession number(s).

The V. penaeicida TUMSAT-NU1 scaffolds are available at DDBJ/EMBL/GenBank under the accession numbers BFAQ01000001 to BFAQ01000100. The raw Illumina read data can be found in the DDBJ Sequence Read Archive with the accession number DRA006460.
